# From Prediction to Generation: Artificial Intelligence Reconstructing the Paradigm of Protein Glycosylation Research

**DOI:** 10.3390/ijms27209032

**Published:** 2026-10-12

**Authors:** Junhong Zhu, Shuang Huang, Nannan Zhang

**Affiliations:** 1 Center for Medical Genetics, West China Second University Hospital, Sichuan University, Chengdu 610041, China; 19332457692@163.com (J.Z.); shuangwwh47@163.com (S.H.); 2 Key Laboratory of Birth Defects and Related Diseases of Women and Children, Sichuan University, Ministry of Education, Chengdu 610041, China; 3 West China School of Basic Medical Sciences and Forensic Medicine, Sichuan University, Chengdu 610041, China; 4 National Center for Birth Defect Monitoring, West China Second University Hospital, Sichuan University, Chengdu 610041, China; 5 State Key Laboratory of Biotherapy, Collaborative Innovation Center of Biotherapy, Sichuan University, Chengdu 610041, China

**Keywords:** protein glycosylation, glycan language, artificial intelligence, protein language model, glycosite prediction, glycoprotein structure, glycoengineering

## Abstract

Among post-translational modifications, protein glycosylation is distinguished by its abundance and structural intricacy. Unlike protein biosynthesis, glycan assembly follows no template; it reflects glycosyltransferase/glycosidase competition, sugar-donor availability, and organelle-enforced compartmentalization, yielding heterogeneous glycoforms that confer functional versatility but complicate analysis. Three bottlenecks persist: stereochemical control in chemical glycosylation, ambiguous assignment of occupancy sites, and reproducing native conformations of fully glycosylated proteins. Adopting the “glycan language” framework, we survey how AI addresses each, emphasizing the systems on which it has been tested. In synthesis planning, GlycoPredictor uncovered anomeric-selectivity rules such as the anti-nitrile and C2-azido α-directing effects, while Bayesian optimization discovered lithium-salt-directed stereoselective glycosylation; for site identification, ESM-2 with AlphaFold2-derived structural features improves N- and O-glycosite prediction, and DeepGlycanSite maps carbohydrate-binding interfaces. For structure and dynamics, AlphaFold3 and multimodal cryo-electron microscopy (cryo-EM), hydrogen–deuterium exchange mass spectrometry, and molecular dynamics (MD) modeling move glycoproteins toward conformational ensembles, as in the AI-assisted dissection of the MCTS1–JAK2–IFN-γ axis. In glycomics, deep learning resolves intact glycopeptides and de novo glycan topology and has defined Fc N-glycan signatures of immunoglobulin G in autoimmune and malignant disease. Because glycan language models link sequence and structure to function, they are becoming clinically actionable: they prioritize candidate variants in congenital disorders of glycosylation and stratify immune disorders and tumors by aberrant glycosylation. Remaining obstacles include scarce structure-linked datasets, encoding of branched topologies, multimodal fusion, and interpretability.

## 1. Introduction

Protein glycosylation—the covalent attachment of mono- or oligosaccharides to specific amino acid residues, catalyzed by glycosyltransferases and subsequently remodeled by glycosidases—occurs primarily as N- and O-glycosylation. As one of the most widespread and chemically complex post-translational modifications, it governs protein folding, signal transduction, immune recognition, and intercellular communication, and its dysregulation is closely associated with cancer and neurodegenerative disease.

Glycan biosynthesis is not confined to a single compartment, nor is it confined to the secretory pathway. Along the endoplasmic reticulum (ER)–Golgi axis, initiation, processing, and maturation are distributed across the ER, the ER–Golgi intermediate compartment (ERGIC), the Golgi stack, and the trans-Golgi network (TGN), with glycosyltransferases and glycosidases partitioned into distinct subcompartments [1]. Within this secretory pathway, the ER also initiates a set of non-mucin O-glycans: O-mannose by the POMT1/POMT2 complex, O-fucose on EGF-like repeats by POFUT1, O-glucose by POGLUT1, and C-mannosylation of tryptophan residues by DPY19L1/DPY19L3 [2]. Separately, glycosylphosphatidylinositol (GPI) anchors are assembled in the ER and attached to the C-terminus of target proteins. Beyond this canonical N-glycan route, two further compartments contribute. In the nucleocytoplasm, O-GlcNAc is installed by O-GlcNAc transferase (OGT) and removed by O-GlcNAcase (OGA); it is not elongated into complex chains but cycles dynamically on serine and threonine residues of nuclear, cytoplasmic, and mitochondrial proteins [3]. At the cell surface and in endosomes, glycans undergo editing outside the secretory pathway: NEU1 can translocate to the plasma membrane and desialylate surface receptors, and endocytic recycling allows re-sialylation and remodeling of internalized glycoproteins [4]. A further point deserves emphasis: Golgi glycosyltransferases are not statically positioned. They continuously recycle between cisternae and the ER via coat protein complex I (COPI)-coated vesicles, and their steady-state distribution—and hence the glycan structures they produce—depends on this trafficking [5]. This dynamic localization is one reason glycan structure emerges from enzyme competition and substrate availability rather than from a fixed assembly line.

The two major forms of glycosylation differ in a way that matters here. N-glycosylation begins co-translationally in the ER lumen. A 14-sugar precursor, Glc_3_Man_9_GlcNAc_2_, is assembled on a dolichol pyrophosphate carrier and transferred en bloc to asparagine within the Asn-X-Ser/Thr sequon (X ≠ Pro); the glycan is then remodeled during Golgi transit [2]. O-glycosylation encompasses several classes that differ in initiating compartment and enzyme family—mucin-type O-GalNAc in the Golgi, O-GlcNAc in the nucleocytoplasm, and O-mannose, O-fucose, O-glucose, and C-mannose in the ER. Among these, mucin-type O-GalNAc is initiated post-translationally in the Golgi by the uridine diphosphate (UDP)-N-acetylgalactosamine:polypeptide N-acetylgalactosaminyltransferase (GALNT) family, which transfers N-acetylgalactosamine (GalNAc) to serine or threonine on folded substrates. The critical asymmetry is that N-glycosylation is guided by a recognizable sequon whereas mucin-type O-glycosylation—the class on which prediction effort has concentrated—has no universal consensus motif, a distinction that largely explains why O-glycosite prediction has proven far less tractable and has become a primary target for machine learning.

This structural complexity, compounded by the absence of a nucleic-acid template for glycan assembly and by the motif-free character of mucin-type O-glycosylation, gives rise to three long-standing difficulties: stereochemical control in chemical glycosylation, confident localization of modification sites, and accurate modeling of fully glycosylated protein conformations. Conventional experimental and computational strategies, despite considerable progress, still show limited generalizability, insufficient throughput, and inadequate accuracy when confronted with physiologically relevant systems.

**Figure 1:**
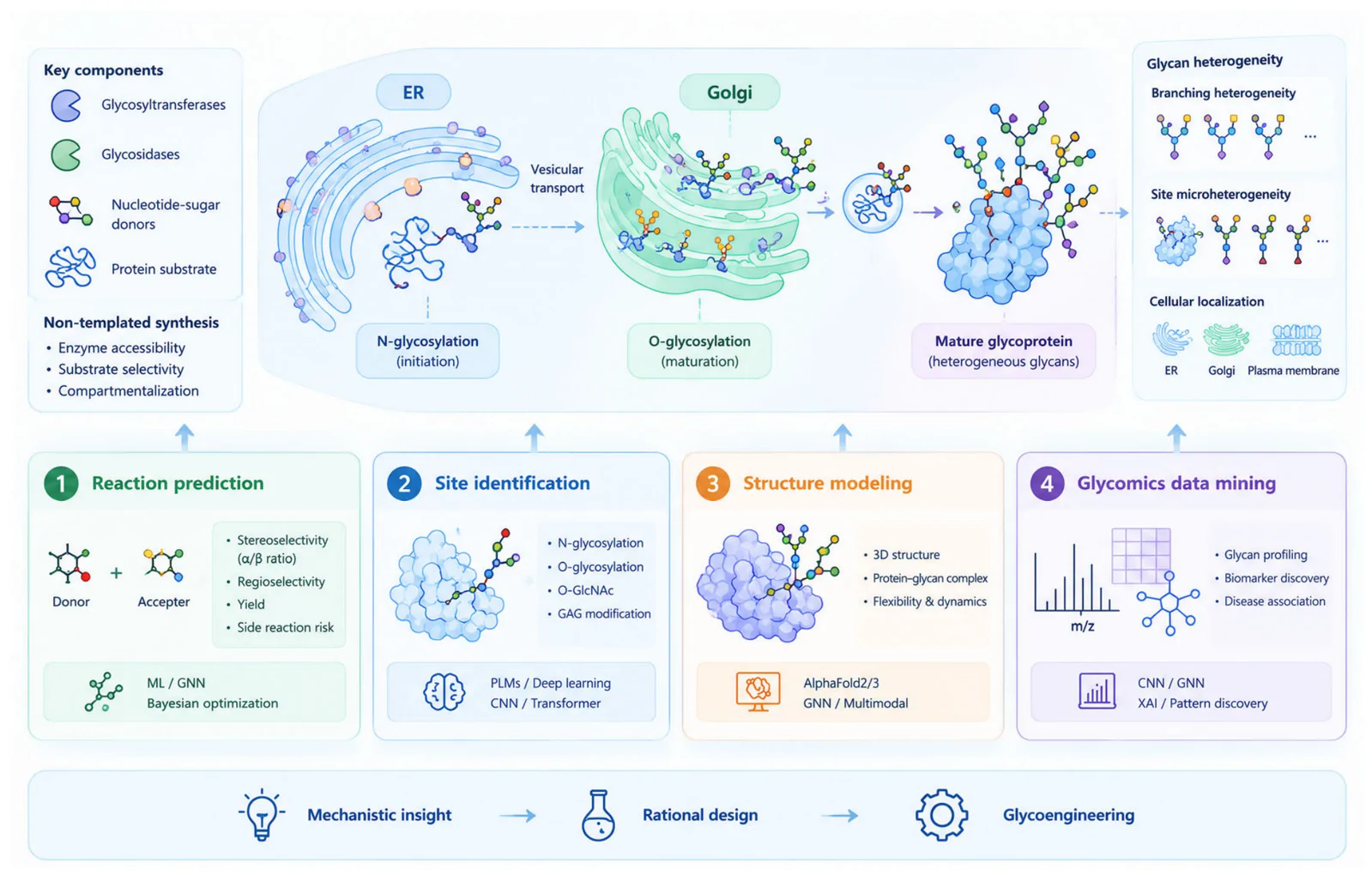
AI-driven protein glycosylation research landscape. The upper panel depicts the glycosylation machinery along the endoplasmic reticulum (ER)–Golgi axis, where glycosyltransferases and glycosidases act on protein substrates using sugar-nucleotide donors to install N- and O-glycans, generating mature glycoproteins bearing heterogeneous, branched glycan structures. The lower panel summarizes four interconnected areas in which AI tools intervene—prediction of glycosylation reactions, identification of glycosylation sites, structural modeling of glycoproteins, and analysis of glycomics data—and traces their progression from mechanistic insight toward rational design and glycoengineering and different colors represent distinct glycan structures.

Artificial intelligence is beginning to change this picture. Machine learning models now predict anomeric selectivity and reaction yields in glycosylation chemistry with useful precision [6]; protein language models have sharpened glycosite detection [7]; and structure prediction tools of the latest generation, most notably AlphaFold3 (AF3), have extended their reach to protein–glycan complexes [8]. AF3 is the first unified end-to-end framework able to model proteins, nucleic acids, small molecules, ions, and modified residues within a single architecture, and its release permits customizable glycan input. Systematic evaluation, however, reveals that this capacity is conditional: input format strongly affects stereochemical fidelity, standard confidence metrics fail to detect anomeric or epimeric errors, and outputs are static snapshots that do not capture glycan conformational dynamics [9]. Glycoenzyme–substrate complexes such as MAN1A1 with its Man9 substrate can nevertheless be reproduced to sub-angstrom accuracy. These findings imply that AF3 models require independent stereochemical validation rather than reliance on built-in confidence scores and that glycan conformational dynamics must be recovered by other means. Multimodal strategies are therefore essential—cryo-electron microscopy, hydrogen–deuterium exchange mass spectrometry, and molecular dynamics simulations are needed to move glycoprotein modeling from static structures toward conformational ensembles. Beyond prediction, AI is also acting as an engine for autonomous exploration: Bayesian optimization can probe uncharted reaction conditions and uncover new stereochemical patterns, hinting at a genuine capacity to assist scientific discovery (Figure 1).

Taken together, these developments point to deepening AI integration across glycochemistry, glycomics, and glycoprotein structural biology. Yet the relevant work remains dispersed across research groups, and a coherent review structure has yet to materialize. Deep learning is the common thread, applied throughout to extract biological principles from glycan chains, protein sequences, and structural frameworks, supporting both interpretation and engineering of glycosylation-related properties. In this review, we survey recent advances across these four areas—prediction of glycosylation reactions, identification of glycosylation sites, structural modeling of glycoproteins, and analysis of glycomics data—before considering the obstacles ahead and sketching potential avenues forward.

## 2. AI in Glycosylation Reaction Prediction

This section surveys AI-driven advances in glycosylation reaction prediction, with emphasis on stereoselectivity control and reaction condition optimization.

### 2.1. Challenges in Stereoselective Glycosidic Bond Formation and Limitations of Traditional Approaches

**Figure 2:**
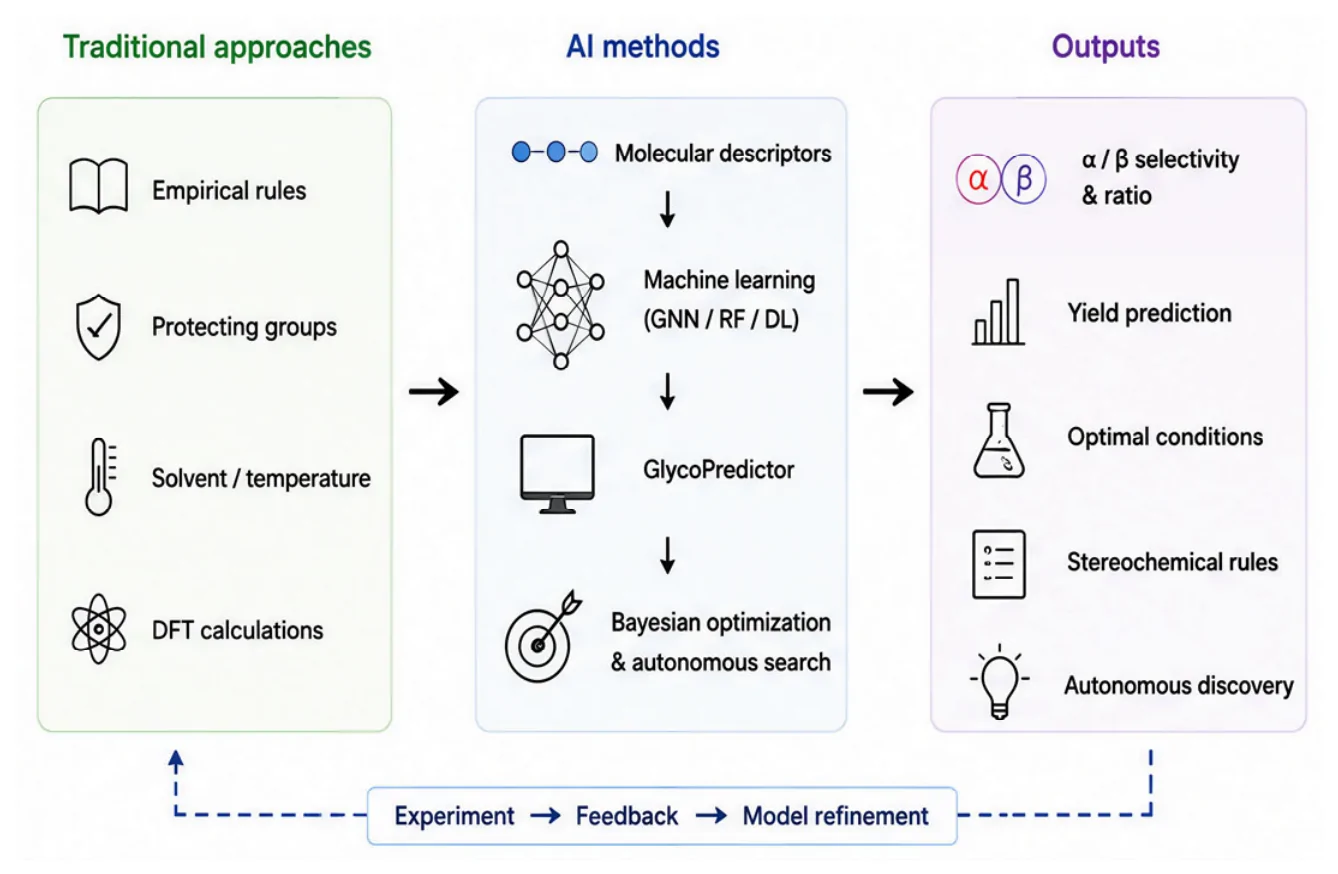
Al for glycosylation reaction and stereoselectivity prediction. Traditional approaches—empirical rules, protecting group strategies, solvent/temperature tuning, and DFT calculations—are contrasted with modern AI methods that employ molecular descriptors alongside machine learning algorithms (e.g., SVM, random forests, deep learning) and dedicated tools like GlycoPredictor and Bayesian optimization for autonomous condition search. The outputs span α/β selectivity and ratios, yield forecasts, optimal reaction parameters, stereochemical principles, and even de novo discovery of reaction conditions, illustrating how data-driven strategies are reshaping this chemistry.

In glycosylation chemistry, the overriding challenge is to gain reliable stereochemical control at the anomeric carbon. The stereochemical outcome depends on a multidimensional set of variables—including the nature of the donor and acceptor and the protecting group pattern—and has long been regarded as a “black box” [10]. Traditional approaches have relied on empirical rules and quantum chemical computations, but both have clear limits. Among the empirical guidelines, neighboring-group participation is the most widely applied: the presence of a participating acyl group at the C2 position of the donor effectively promotes the formation of 1,2-trans glycosidic bonds. For substrates like 2-deoxy sugars and sialic acid derivatives, however, stereoselectivity still responds to solvent, temperature, ionic effects, and additional parameters, so capturing it with a single set of rules remains difficult. Density functional theory (DFT) can resolve transition-state geometries and explain individual reaction outcomes, but it is computationally expensive, and extending it to a general predictive framework across diverse substrate classes has not been achieved. Reported DFT studies typically address one donor–acceptor pair at a time—for example, the mechanistic analysis of glycosylation by Fu, Bernasconi, and Li [11]—and their conclusions do not transfer directly to other protecting-group or solvent regimes. As a result, until recently the prediction of stereoselectivity relied mainly on cumulative experience and trial-and-error optimization, creating a clear opening for data-driven approaches (Figure 2).

### 2.2. Machine Learning-Driven Prediction of Stereoselectivity

In recent years, AI-driven glycosylation prediction has progressed from pattern mining and high-accuracy forecasting toward the autonomous discovery of new reactions. Three representative contributions are discussed below.

#### 2.2.1. GlycoPredictor: Mining Chemical Rules from the Literature

Faurschou et al. developed GlycoPredictor and trained it on a corpus of approximately 10,434 glycosylation reactions extracted from CAS (2010–2015), supplemented by the 734-reaction Barresi–Hindsgaul dataset, covering O-, S-, and N-glycosylations as well as pyranose and furanose donors. The model predicts the major anomer, the minor anomer, and their ratio simultaneously [6]. Using interpretable decision-tree analysis, the model quantified the contributions of individual molecular features to stereoselectivity and identified several rules that had not been systematically characterized before, including the anti-nitrile effect of 2-deoxy sugars in acetonitrile, the α-directing character of DMT/DBT triazine-based leaving groups, and the α-directing influence of a C2-azido substituent. These predictions were subsequently validated by experiments: the authors synthesized DMT donors and confirmed the α-selectivity of 2-deoxy sugars in acetonitrile. Beyond random train–test splitting, the model was evaluated using a publication split, in which entire publications were held out to simulate prediction of previously unseen methods. Regression performance dropped sharply: R^2^ fell from 0.57 under random splitting to 0.11 under the publication split, indicating that the model explains only 11% of the variance when extrapolating to new publications. Classification of the major anomer, however, was less affected under the same split, suggesting that the model retains some transferable discriminative ability even when absolute yield or ratio prediction fails. This gap is itself informative—it quantifies how much of the reported performance depends on chemical similarity between training and test entries, and it highlights cross-publication generalization as an unresolved challenge.

#### 2.2.2. Bayesian Optimization: Autonomous Discovery of New Reactions

Building on the predictive models, Faurschou and Pedersen turned to Bayesian optimization to carry out an autonomous exploration of glycosylation reaction conditions [12]. In this framework, the glycosylation reaction is treated as an unknown function, and the high-dimensional reaction space is iteratively searched without relying on any prior reaction data. This search led to the identification of a novel lithium-salt-directed stereoselective glycosylation strategy. Partial dependence plots further indicated how lithium concentration and counterion type affect stereoselectivity, thereby giving the optimization a measure of chemical interpretability. What this study demonstrates is that Bayesian optimization can identify useful reaction conditions outside the training distribution; whether the lithium-salt effect constitutes a general stereochemical principle or a substrate-specific observation remains to be tested across a broader range of donors and acceptors.

### 2.3. Current Limitations and Future Perspectives

Despite the progress described above, several limitations remain, and they constrain what can currently be claimed for AI in glycosylation prediction.

The first is data. Current models are built on datasets curated from the literature or from standardized experiments. Even the largest reported dataset—10,434 reactions for GlycoPredictor—is small by machine-learning standards, and the distribution of substrates, protecting groups, and reaction conditions within it is uneven. Reported performance metrics therefore reflect the distribution of the training data rather than the chemical space as a whole.

The second is method comparison. Few studies evaluate multiple machine-learning approaches on the same dataset with the same split and the same metrics. Where comparisons exist, they are often confounded by differences in feature representation, dataset size, and validation protocol. In the GlycoPredictor benchmark, the descriptor-based random forest achieved the best overall performance, with area under the curve (AUC) values of 0.89–0.97 depending on the splitting strategy, outperforming both a graph neural network (Chemprop) and a decision tree [6]. This result suggests that, at the current dataset scale, well-tuned descriptor models can remain competitive with graph-based architectures, and it argues against assuming that graph neural networks are inherently superior for glycosylation prediction. More generally, models trained on large curated corpora achieve the highest reported accuracy within their training distribution, but the gap between in-distribution and out-of-distribution performance is rarely quantified, and prospective validation on reactions designed after training remains uncommon. Data leakage, where chemically similar reactions appear in both training and test sets, is difficult to exclude in datasets curated from the literature and is seldom addressed explicitly. A recent review by Yin et al. systematically surveys AI approaches for predicting stereoselectivity in glycosylation chemistry, covering both descriptor-based and graph-based models, and discusses the challenges of data scarcity, reaction space coverage, and model interpretability [10].

The third is scope. Current models predict the outcome of a single glycosylation step. The assembly of a complex oligosaccharide involves cumulative effects across multiple steps, including remote protecting-group effects, conformational changes, and intramolecular interactions. Predictive frameworks that capture multi-step pathways have not yet been demonstrated.

The fourth is integration with experiment. Deep integration of AI models with automated synthesis platforms remains at an early stage. Closed-loop cycles combining high-quality reaction databases, automated experimentation, and active learning have been proposed but not yet realized at scale for glycosylation.

Taken together, glycosylation prediction is moving from an empirical pursuit toward a data-driven discipline, but the field is not yet at the point where AI can be said to “discover new chemical principles” in a general sense. The demonstrated achievements are narrower and more concrete: machine-learning models can reproduce and rationalize stereochemical outcomes within defined substrate classes, and Bayesian optimization can identify useful reaction conditions outside the training set. Extending these results to broad, generalizable prediction and design in complex molecular settings remains an open objective.

## 3. AI in the Intelligent Identification of Glycosylation Sites

### 3.1. Core Challenges and Limitations of Traditional Identification Methods

Glycosylation site mapping still relies chiefly on mass spectrometry, but high costs, limited throughput, and reliance on expert-driven interpretation have hindered the generation of high-quality annotated datasets. Verified entries in current databases are heavily biased toward canonical N-glycosylation, whereas reliable data for O-glycosylation—where a well-defined sequence motif is lacking—low-stoichiometry O-GlcNAc modifications and glycosaminoglycan-related events are far sparser. On top of that, discrepancies in data formats across experimental platforms, the frequent absence of detailed glycan structural information, and the lack of uniform quality control standards further impede the assembly of high-confidence training sets.

Before the rise of deep learning, glycosylation site prediction leaned chiefly on sequence motif recognition, handcrafted feature engineering, and traditional machine learning methods. In the case of N-glycosylation, predictions typically hinged on the classic Asn-X-Ser/Thr (X ≠ Pro) motif; in reality, however, whether a site actually becomes modified is shaped by the protein’s structural environment and the cellular context, so relying on sequence rules alone inevitably misses non-canonical sites. For O-glycosylation, which lacks a universal consensus sequence, the predictive reach of such approaches is even more limited. These earlier strategies also drew largely on primary sequence features—amino acid composition, physicochemical properties, and the like—and were poorly suited to incorporating three-dimensional information such as site accessibility, local conformation, and the recognition landscape of the relevant transferases. These models, meanwhile, used shallow architectures—support vector machines (SVMs), random forests, and naïve Bayes—whose restricted capacity struggled with the nonlinear relationships and residue cooperativity often governing glycosylation. It is precisely this limited generalizability under complex conditions that prompted the move to deep learning.

### 3.2. The Shift from Traditional Machine Learning to Deep Learning

#### 3.2.1. Traditional Machine Learning Methods

Early efforts to computationally identify glycosylation sites grew out of statistical modeling of specific sequence motifs and physicochemical properties. As noted by Alkuhlani et al., these initial prediction strategies fell broadly into two groups: rule-based recognition built on the classic N-glycosylation sequon, and classifiers such as support vector machines and random forests [13].

Akmal and coworkers systematically assessed machine-learning models for N-linked glycosylation site prediction, observing that while approaches relying on hand-crafted descriptors—including amino acid composition, physicochemical properties, and evolutionary information—can reach reasonable accuracy, their success depends heavily on the feature design, and they often underperform at non-canonical sites [14]. A further complication is the severe imbalance between experimentally confirmed positive and negative examples; large numbers of unannotated sites cannot simply be assumed to be negative, making it difficult for conventional supervised learning to establish a trustworthy decision boundary. Working within a positive-unlabeled learning framework, Mahesworo et al. explored multilayer perceptron architectures and, through careful tuning of the loss function and sampling strategy, boosted predictive power in data-scarce settings—a finding that also underscores the intrinsic limitations of traditional models when faced with complex biological data [15].

#### 3.2.2. Deep Learning Models

The arrival of deep learning redirected glycosylation site prediction from manual feature engineering toward automated representation learning. The central advantage is that deep neural networks can learn hierarchical feature representations directly from raw sequences, freeing the models from the hard-coded rules that dominated earlier work.

Over the course of this evolution, convolutional neural networks (CNNs) were first deployed to capture local sequence motifs associated with glycosylation, whereas recurrent neural networks (RNNs) and long short-term memory (LSTM) architectures later augmented the capability for modeling long-range residue interactions. Hu’s group put forward N-GlycoPred, an architecture that fuses a convolutional network with a bidirectional LSTM to simultaneously exploit local sequence context and global sequence information; the model delivered stable N-glycosylation site prediction performance across several benchmark datasets [16].

For O-glycosylation, where a rigid consensus motif is absent, Pham et al. developed HOTGpred. By integrating pretrained protein features with a multi-stage feature selection strategy, the method raised the accuracy of predicting human O-linked threonine glycosylation sites, showing that deep learning can extract meaningful discriminative signals even when sequence constraints are weak [17]. As with N-glycosylation, the reported gains are difficult to compare directly across studies, because the studies differ in negative-set construction and evaluation protocol and a common benchmark for glycosylation site prediction has not yet been adopted.

#### 3.2.3. Protein Language Models and Multimodal Learning

The recent adoption of Transformer-based protein language models (PLMs) has given new momentum to glycosylation site prediction. Through self-supervised training on large-scale protein sequence collections, these models acquire coevolutionary information, structural biases, and functional limitations, in turn delivering richer sequence encodings for subsequent tasks. PTG-PLM, introduced by Alkuhlani et al., marked the first application of pretrained PLMs to glycosylation-related tasks, showing that embedding-based features can improve post-translational modification site prediction relative to hand-crafted descriptors [7]. EMNGly subsequently reported that language model-derived features improve N-glycosylation site identification over sequence-only baselines [18]. In both cases, however, the evaluation was carried out on datasets assembled by the authors, and cross-study comparison is limited by differences in negative-set construction and performance metrics. These advances subsequently revealed that sequence information alone is often insufficient for capturing the spatial and local microenvironmental constraints governing glycosylation site formation. Structural information has therefore been progressively woven into glycosylation site predictors, spurring the development of sequence-structure multimodal fusion strategies. CoNGlyPred represents a concrete implementation of this strategy. The model combines deep sequence representations extracted by ESM-2 with three-dimensional features—including residue depth, secondary structure composition, and solvent-accessible surface area—computed from AlphaFold2-predicted structures, and integrates them through a graph network with co-attention mechanisms [19]. Adding structural features improved performance over a sequence-only baseline, particularly for sites with sterically constrained occupancy. A caveat is that the structural features are derived from predicted rather than experimental structures, so errors in the predicted fold propagate into the site predictor; the extent to which this affects performance on proteins with low-confidence AlphaFold2 models has not been systematically assessed.

### 3.3. Structure-Aware AI and Graph Neural Networks in Glycosylation Research

Glycosylation is shaped not only by the local sequence environment but also critically by the three-dimensional conformation of the protein and the spatial topology of the glycan chain. Incorporating structural information into AI models has therefore emerged as a key direction for improving predictive performance.

The branched architecture of glycans and the interfaces of protein–carbohydrate complexes are inherently non-Euclidean data structures—precisely the kind that traditional machine learning methods struggle to represent efficiently. Graph neural networks (GNNs) offer a natural route. Joeres and Bojar used higher-order message-passing for glycan representation learning, showing that GNNs can encode branching and linkage patterns that linear representations cannot capture directly [20]. This work is primarily a representation-learning study rather than a benchmark against other glycan encodings on a common task, so its practical advantage over sequence- or fingerprint-based representations remains to be quantified. In the study of protein–carbohydrate interactions, the DIONYSUS database curated by Gheeraert and colleagues houses a large collection of structural data on protein–carbohydrate interfaces, providing a valuable resource for binding-site analysis [21]. Structure-aware GNN approaches to carbohydrate-binding site prediction, including DeepGlycanSite [22], are discussed in Section 4.1.2 in the context of glycoprotein complex modeling.

### 3.4. Deep Integration of AI with Mass Spectrometry Data

#### 3.4.1. Advances and Challenges in Mass Spectrometry

Mass spectrometry-based glycoproteomics has become an indispensable tool for glycosylation site identification and glycan structural characterization. Yet, the confident identification of intact glycopeptides and the discrimination of glycan isomers remain major bottlenecks in high-throughput workflows. Cao and colleagues noted that while mass spectrometry can supply information on glycan composition, branching patterns, and linkages—and has been widely applied to disease biomarker discovery and glycoform profiling of therapeutic antibodies—the enormous search space of complex glycopeptides and the diversity of fragmentation modes are steadily pushing conventional database search strategies toward their performance ceilings [23]. As a result, the primary limitation in glycoproteomics has shifted from data generation to data interpretation, opening clear avenues for artificial intelligence intervention.

#### 3.4.2. AI-Assisted Mass Spectrometry Data Analysis

Artificial intelligence is advancing glycoproteomics on two main fronts: glycopeptide identification and glycan structural elucidation. For glycopeptide identification, the pGlyco3 search engine developed by Zeng and colleagues employs a glycan-first strategy that decouples glycan and peptide searching and combines ion annotation with a dedicated scoring algorithm to enable the efficient identification of intact N- and O-glycopeptides as well as modified glyco-units [24]. Its false-discovery control relies on decoy-based estimation, which is well established for peptides but less thoroughly validated for glycan moieties.

On the side of glycan structure prediction, Urban et al. introduced CandyCrunch, a method that uses dilated residual neural networks to directly ingest liquid chromatography–tandem mass spectrometry (LC-MS/MS) data and perform end-to-end glycan structure prediction. By bypassing manual annotation, CandyCrunch predicts glycan structure from raw LC–MS/MS data in seconds, with a top-1 accuracy of 90.3% on an independent test set [25]. The model was trained on nearly 500,000 annotated spectra from more than 2000 glycomics experiments. Because CandyCrunch is formulated as a multiclass classifier, it can only assign structures represented among its training classes; extension to genuinely novel structures requires generative formulations such as GlycoBART [26], or biosynthetic-network completion as implemented in the associated glycowork pipeline [25]. Accuracy is correspondingly lower for rare glycoforms and non-canonical modifications.

Addressing the persistent challenge of glycan isomer differentiation, the group led by Zhu further explored the potential of coupling ion mobility mass spectrometry (IM-MS) with artificial intelligence. They argue that deep learning is well positioned to extract isomer-discriminating features from high-dimensional spectra and drift-time information, enabling the precise distinction of complex glycan structures [27].

### 3.5. Challenges and Future Perspectives

Although AI has markedly accelerated progress in glycosylation research, its further application remains constrained by limited data resources and model generalizability. A major obstacle remains the scarcity of well-annotated data. Glycosylation shows marked specificity across tissues, species, and spatiotemporal contexts, and the expense of experimental validation has resulted in an unequal representation of glycosylation types in existing databases. N-glycosylation is overrepresented relative to O-glycosylation, and within O-glycosylation, mucin-type O-GalNAc is overrepresented relative to O-GlcNAc and ER-initiated O-glycans. This imbalance limits the applicability of models to complex physiological settings and biases predictions toward the most frequently annotated classes.

A second concern is evaluation practice. Most predictors are assessed on datasets assembled by the same group that developed the model, with negative sets constructed differently across studies, and few are tested on fully independent datasets. Overfitting to dataset-specific features and data leakage between training and test splits are difficult to exclude under these conditions and are rarely addressed explicitly. The field would benefit from a shared benchmark with fixed positive and negative sets and standardized metrics, similar to what has been developed for other post-translational modification prediction tasks.

A third concern is interpretability. Many of the best-performing models are deep networks whose predictions cannot be traced back to specific sequence or structural features. For glycosylation site prediction, interpretability matters because the goal is not only to classify sites but to understand why a given site is or is not modified. Attention-based and feature-attribution methods offer partial answers, but their outputs require careful validation and should not be over-interpreted.

A fourth concern is computational cost. Training protein language models and structure-aware predictors requires substantial graphics processing unit (GPU) resources, and inference on proteome-scale datasets can be expensive. This limits routine use in laboratories that lack dedicated computational infrastructure and constrains the practical deployment of the largest models.

Looking forward, a key priority is to create standardized databases and develop multimodal systems that can integrate information across diverse sources—spanning sequence, structure, mass spectrometry, and imaging. Strengthening model clarity is equally important for moving AI-based methods into clinical and pharmaceutical settings. Deep learning, graph networks, generative models, and related techniques together hold promise for improving predictive performance and uncovering mechanistic insights, but the field is not yet at the point where these methods can be described as routinely reliable on independent data. Progress will depend as much on data infrastructure and evaluation standards as on model architecture.

## 4. Structural Modeling of Glycoproteins

The three-dimensional architecture of glycoproteins forms the essential basis for understanding how glycosylation modulates protein folding, molecular recognition, and biological function. Glycoprotein structure determination, unlike that of unmodified proteins, faces distinct challenges—glycan flexibility, branching heterogeneity, and dynamic glycan–protein interfaces—which hinder classical experimental and computational approaches from fully capturing their true conformational states. The past few years have seen considerable progress in AI-based structure prediction, providing fresh theoretical frameworks and computational tools for glycoprotein modeling and moving the field from static predictions toward multiscale, multimodal, and dynamic conformational descriptions.

### 4.1. AI-Driven Glycoprotein Structure Prediction and Complex Modeling

Deep learning has substantially improved protein structure prediction, although its performance on glycoproteins remains uneven. Individual models differ in design principles and applicability, and no universal framework yet handles all glycoprotein systems.

#### 4.1.1. Architectural Breakthroughs in Monomer and Multimer Structure Prediction

Deep learning models, most notably AlphaFold and RoseTTAFold, have achieved a transformative leap in predicting high-accuracy three-dimensional structures directly from protein sequences. AlphaFold2 uses its Evoformer to combine multiple sequence alignments with pairwise residue data, applies attention mechanisms to derive evolutionary signals, and feeds these into the structure module. This module then produces 3D conformations satisfying geometric constraints—yielding a direct mapping from sequence to structure [28]. RoseTTAFold, by contrast, adopts a three-track architecture that processes sequence, distance maps, and three-dimensional coordinates in parallel, giving it distinct advantages in computational efficiency and adaptability to complex systems [29].

Following these foundations, systematic advances have been achieved in modeling protein complexes. AlphaFold-Multimer, from the DeepMind group, enhances multi-chain representation and integrates cross-chain invariant point attention, allowing effective interface prediction for both homomeric and heteromeric complexes. Its primary strength is its use of co-evolutionary data to infer spatial constraints between subunits [30]. Meanwhile, RoseTTAFold All-Atom (RFAA), developed by the Baker group at the University of Washington, extends the modeling reach to entire biomolecular systems—proteins, nucleic acids, small molecules, metal ions, and covalent modifications—through an all-atom molecular representation coupled with a deep learning architecture [31]. To tackle the assembly of large complexes, CombFold uses a hierarchical assembly strategy that progressively constructs multi-subunit architectures guided by structure prediction scores, markedly boosting prediction efficiency for very large systems [32]. Altogether, these tools provide a methodological base for glycoprotein complex modeling.

Interpreting these outputs requires distinguishing confidence metrics from accuracy metrics. Modern structure prediction tools commonly report per-residue confidence scores such as pLDDT (predicted local distance difference test), which estimates the reliability of each residue’s local environment on a 0–100 scale; values above 90 indicate confident backbone prediction, whereas those below 50 often correspond to disordered regions. Because pLDDT reports only on local geometry, the correctness of relative domain or chain placement is given separately by PAE (predicted aligned error), which estimates the expected positional error in ångströms for each residue pair. Both are model-internal confidence estimates and do not by themselves guarantee agreement with experimental structures. Accuracy metrics instead require an independent reference structure, most commonly RMSD (root-mean-square deviation between corresponding atoms after optimal superposition), whose magnitude depends on which atoms are compared and whether superposition is global or restricted to a binding site. Confusing the two classes is consequential: a model may carry high pLDDT and low PAE yet deviate substantially from experiments in RMSD—a dissociation that can be especially problematic for glycans, as discussed in Section 4.1.2.

#### 4.1.2. Unique Challenges and Strategies in Glycoprotein Complex Modeling

When applied to glycoprotein complexes, the general-purpose models described above face three distinct challenges. To begin with, glycan chains exhibit high flexibility and broad branching heterogeneity, which creates substantial conformational diversity in physiological settings. In addition, induced fit effects frequently arise during complex formation, so models must accommodate the dynamic conformational shifts linked to binding—something that current mainstream approaches still struggle to accomplish. Besides, complexes that rely on weak or transient interactions tend to feature highly flexible interfaces and pronounced conformational heterogeneity, and their prediction accuracy is consistently and markedly lower than that of stable complexes.

To address these limitations, researchers have pursued advances along several fronts. At the level of carbohydrate-binding site prediction, He et al. developed DeepGlycanSite, which fuses protein geometric structure with evolutionary information through an equivariant graph neural network and reports high accuracy for carbohydrate-binding site prediction on its benchmark dataset, though glycoprotein-specific performance is less established [22]. At the level of overall complex modeling, a new generation of generative models, most notably AlphaFold3, has for the first time brought glycans, post-translational modifications, proteins, and ligands together within a unified end-to-end modeling framework. Its published examples include the glycosylated globular portion of an EXTL3 homodimer (Protein Data Bank (PDB) 7AU2; mean pocket-aligned RMSD, 1.10 Å) and the heavily glycosylated human coronavirus OC43 spike protein bound by neutralizing antibodies (PDB 7PNM, 4665 residues), establishing that glycans can be treated as first-class molecular entities rather than as post hoc decoration [8]. Carbohydrate ligand prediction accuracy still lags behind that of proteins and nucleic acids, although these models have already demonstrated promise in forecasting binding modes and mapping interaction sites. Systematic evaluation, however, shows that this capacity is conditional [9]. Input representation is decisive: simplified molecular-input line-entry system (SMILES) and user-defined Chemical Component Dictionary (CCD) inputs frequently produce stereochemical errors, including misassignment of the galactose C2 hydroxyl and inverted α/β anomeric configurations, whereas Chemical Component Dictionary building blocks linked through “bondedAtomPairs” syntax yield stereochemically valid models [9]. Equally important, the model’s own confidence measures—pLDDT, PAE, interface predicted TM-score (ipTM), and ranking score—fail to detect anomeric or epimeric errors, so stereochemically incorrect glycans may still receive high scores [9]. Where the input is specified correctly, the results can be accurate: AF3 reproduced the Michaelis complex of MAN1A1 with its Man9 substrate, as well as MGAT2, ST6GAL1, GPI anchors, and the glycosylated Siglec-2 (CD22) ectodomain [8]. At the level of functional mechanism dissection, Bohlen et al. combined AI-predicted structures with biochemical validation to uncover the molecular mechanism by which *MCTS1* deficiency modulates *JAK2* translation reinitiation and disrupts IL-23-dependent IFN-γ immune signaling [33]. This example involves a non-glycosylated signaling axis, and its inclusion is intended to illustrate the general workflow of combining AI-predicted structures with biochemical validation, not to claim that the same mechanism operates in glycosylated systems.

In the especially valuable context of antibody–glycan antigen interactions, current models still encounter major bottlenecks. Compared with protein–protein complexes, training data for antibody–glycan antigen prediction are severely scarce. Furthermore, glycan antigens experience substantial conformational changes in physiological settings, and the binding process entails complex dynamic elements—such as sugar ring conformational shifts, hydrogen-bond network rearrangements, and multivalent interactions. Future work should combine conformational sampling, molecular dynamics simulations, and experimental structural data toward a more accurate dynamic modeling framework. Quantitative comparison of AF3 against experimental glycoprotein structures remains limited; the examples above are illustrative rather than benchmarked across a common test set.

#### 4.1.3. Multimodal Information Fusion and Dynamic Conformational Modeling

Building on this shift, the OneProt framework introduced by Flöge et al. exemplifies multimodal fusion: by aligning multiple information sources—sequences, multiple sequence alignments, domain annotations, and functional site data—within a shared latent space, it enables synergistic learning across modalities and demonstrates that multimodal feature fusion can markedly enhance protein function prediction [34].

**Figure 3:**
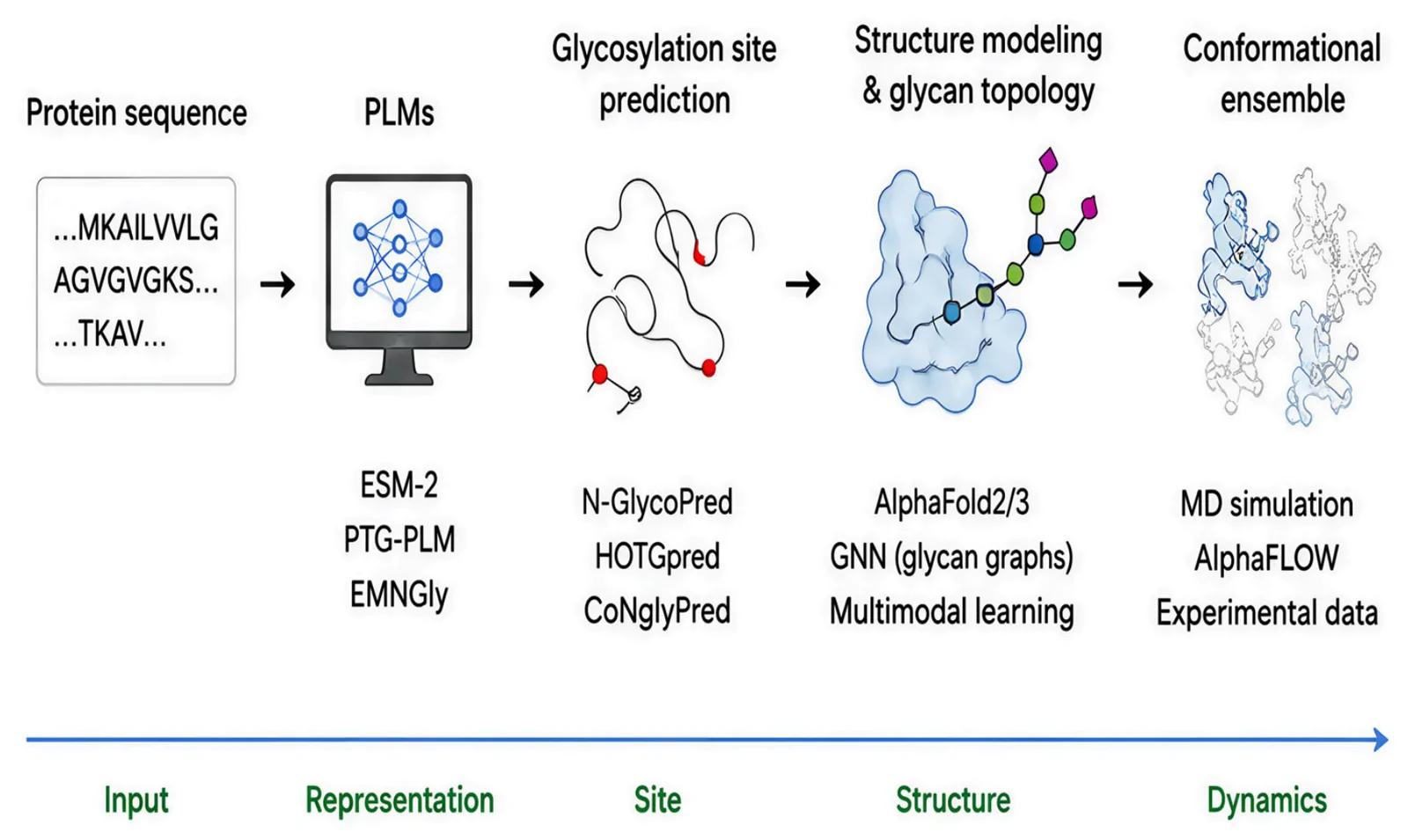
From sequence to structure: Al modeling of glycoproteins. This schematic illustrates the current AI-driven pipeline that connects protein primary sequence to three-dimensional conformation and dynamic behavior. Starting from input sequences, protein language models including ESM-2, PTG-PLM, and EMNGly generate rich representations that feed into glycosylation site predictors such as N-GlycoPred, HOTGpred, and CoNglyPred. The identified sites then guide structural modeling using AlphaFold2/3 and graph neural networks for glycan topology, with multimodal learning further refining the predictions. Moving beyond static structures, the framework incorporates molecular dynamics simulations, AlphaFLOW, and experimental data to sample conformational ensembles, thereby capturing the intrinsic flexibility of glycoproteins. The lower panel depicts representative glycan symbols and peptide backbones, underscoring the molecular complexity addressed by these computational strategies. The colored symbols represent distinct monosaccharide units.

Protein function arises from dynamic conformational transitions rather than from a single rigid shape. AlphaFLOW, introduced by Bowen Jing and coworkers, combines flow matching with AlphaFold-derived structural data for efficient sampling of the protein conformational landscape—capturing metastable states beyond conventional static predictors and offering a fresh computational resource for studying protein dynamics [35]. When paired with molecular dynamics simulations and diffusion models, such generative sampling methods have considerable potential for further probing glycan chain flexibility, conformational rearrangements, and the dynamics that underlie protein–glycan interactions—thus supporting the resolution of the glycoprotein complex modeling challenges noted previously (Figure 3).

The vast majority of predicted structures deposited in the AlphaFold database largely represent probable dominant conformations and cannot fully reflect the dynamic behavior of proteins in solution. For glycoprotein systems, the difficulty is compounded by glycan flexibility, conformational transitions of sugar chains, and glycosylation-induced local structural rearrangements. Accordingly, the goal of structure prediction is shifting from determining a single structure toward describing conformational distributions and their functionally relevant states.

### 4.2. From Static Structure Prediction to Functional Conformational Ensemble Modeling

#### 4.2.1. The Necessity and Challenges of Dynamic Conformational Modeling

In recent years, AI-based generative models have opened up new strategies for dynamic structure prediction. AlphaFLOW, developed by Bowen Jing and colleagues, combines flow-based models with AlphaFold structural representations to sample the protein conformational landscape, capturing metastable conformations that conventional static predictors struggle to describe [35]. AI methods integrating molecular dynamics (MD) simulation data have further improved the capacity to learn dynamic behaviors, yielding predictions that satisfy structural plausibility and mirror potential motional paths and conformational transition routes. Current models, however, still face important limitations. Low-pLDDT regions in AlphaFold outputs, frequently accompanied by elevated PAE within and between the corresponding segments, can signal flexible or disordered stretches, yet it remains challenging to reliably distinguish functionally relevant flexibility from genuinely disordered regions—and glycosylation events frequently occur precisely within these highly dynamic zones [36]. A key direction for future progress will therefore be to improve the resolution of flexible glycoprotein regions by tightly integrating experimental data with dynamic simulations.

#### 4.2.2. Multiscale and Multimodal Integration Facilitates Dynamic Structure Determination

Progress in dynamic conformational modeling hinges on the fusion of information from diverse sources. Molecular dynamics (MD) simulations offer trajectories of protein motion and details of conformational transitions, which act as a critical data basis for AI models to learn dynamical principles. Beyond validating predicted structures, MD simulations can likewise serve as informative priors in training and constraining AI models.

Cryo-electron microscopy (cryo-EM) and nuclear magnetic resonance (NMR) spectroscopy, as experimental tools, provide direct evidence of protein conformational heterogeneity, thus providing experimental constraints for guiding dynamic-state predictions. These data, combined with evolutionary information extracted by protein language models from large-scale sequences, will allow future models to merge sequence, structure, dynamics, and experimental observations within one framework, thereby generating more accurate representations of functionally relevant glycoprotein conformations.

For glycoproteins, such a multimodal strategy carries particular significance. Glycan chain motions, local protein flexibility, and rearrangements at glycan–protein interfaces often jointly govern molecular recognition processes—factors that are difficult to capture with single-structure prediction methods. Furthermore, large-scale reprocessing and analysis of public proteomics data have proven valuable for uncovering hidden post-translational modification events and their structural contexts [37]. By fusing information across different scales, AI models are well positioned to elucidate how glycosylation modulates protein function through its influence on conformational states.

#### 4.2.3. Intrinsically Disordered Regions and Glycosylation-Mediated Functional Regulation

Intrinsically disordered regions (IDRs) represent a formidable challenge in glycoprotein structural modeling. These highly dynamic segments resist description by a single set of coordinates, and their conformational flexibility is often intimately linked to regulation by post-translational modifications. Coskuner-Weber and colleagues have noted that the function of intrinsically disordered proteins (IDPs) depends on a delicate balance among multiple conformational states, necessitating their representation as conformational ensembles rather than as single static structures [38].

IDRs are of particular significance in glycosylation research: these regions tend to be solvent-exposed and are therefore highly susceptible to glycosylation, participating in the modulation of protein stability, molecular recognition, and signal transduction.

Molecular dynamics (MD) simulations are uniquely suited to this problem, and their application to glycosylated systems is expanding. Glycoprotein MD is most commonly performed in GROMACS or NAMD, both of which support the CHARMM and AMBER force field families; for carbohydrate systems, this includes CHARMM36 parameters covering furanose and pyranose rings, glycosidic linkages, and sulfated sugars, as well as GLYCAM06, which is compatible with AMBER protein force fields [39]. The recent extension of the CHARMM Drude polarizable force field to N- and O-linked glycopeptides and glycoproteins is accessible through GROMACS, OpenMM, NAMD, and CHARMM via the Drude Prepper module in CHARMM-GUI, providing an explicitly polarizable framework for carbohydrate–protein systems [40]. However, current force fields remain unable to fully capture CH–π contacts between carbohydrates and aromatic residues, and the impact of this limitation on glycoprotein model accuracy remains uncertain. Enhanced sampling approaches—including Gaussian accelerated MD and Hamiltonian replica exchange—have been applied to glycoproteins, but the resulting non-Boltzmann conformations require careful reweighting, and these methods have not yet been widely adopted for glycosylated systems [41].

Despite these constraints, all-atom MD has already yielded mechanistic insight into glycosylated IDR systems. O-GlcNAc transferase (OGT) modifies thousands of nucleocytoplasmic proteins primarily within IDRs, yet O-GlcNAc sites lack a consensus sequence. A recent study combining ~2.1 ms of all-atom MD with Markov state models and transition-state analysis revealed a noncanonical, multi-step induced-fit recognition mechanism: the SMG9-derived substrate anchors at the Int-D SLiM pocket, then engages an external site at the TPR-GTD interface before reaching the catalytic site [42]. This work exemplifies how conventional MD, coupled with Markov state models rather than enhanced sampling, can resolve the dynamic recognition of flexible, IDR-containing substrates by a glycosyltransferase.

Beyond enzyme recognition, MD has been used to quantify how glycosylation alters IDR–ligand interactions. For the O-mannosylated linker of *Trichoderma reesei* Family 7 cellobiohydrolase, MD simulations validated by experimental binding data showed that increasing glycan density weakens lignin binding and partially shifts preference toward cellulose, while phosphorylated mannose abolishes lignin affinity [43]. In a different context, clustered O-GlcNAc sites in the BRD4 C-terminal IDR attenuate condensate size and increase fluidity, thereby modulating transcriptional outcomes linked to the cell cycle and DNA repair [44]. These examples illustrate that glycosylation does not merely decorate IDRs but quantitatively tunes their conformational ensemble and binding preferences—an effect that MD is uniquely positioned to interrogate.

Coupling MD with AI-based generative models may provide a practical route toward capturing conformational biases under distinct modification states.

### 4.3. The Experiment–Computation Closed-Loop Paradigm

Advances in AI-driven structure prediction are beginning to shift protein research away from the conventional “predict–experimentally validate” model and toward a closed-loop cycle of computational design, experimental feedback, and model refinement. In glycoproteins, because glycosylation site selection, glycan conformational dynamics, and functional regulation are all governed by complex structural factors, close synergy between AI and experimental approaches will be crucial for achieving rational design.

#### 4.3.1. AI-Guided Glycosylation Site Engineering and Protein Design

Artificial intelligence is steering protein engineering from empirical screening toward computationally assisted design. Using the ProteinGym platform, Notin and coworkers systematically benchmarked various models for their ability to predict mutation effects, showing that AI can reliably estimate how single amino acid changes influence protein stability, folding, and function—offering new computational tools for rational protein engineering [45]. ProteinGym is built largely on non-glycosylated proteins, so its conclusions transfer to glycoproteins only by extension; glycosylation-site engineering introduces additional variables—glycan heterogeneity, site occupancy, and glycan–protein interactions—that ProteinGym does not capture. This approach can be readily extended to glycosylation site engineering. With structural prediction models, researchers can simulate—even before experiments—how modifying a glycosylation site or adjacent residues might alter local conformation, surface accessibility, and the glycan’s chemical microenvironment, thereby allowing them to prioritize promising candidates for testing.

Looking further ahead, de novo design approaches are pushing protein engineering from “optimizing existing proteins” toward “designing novel functional proteins”. Kortemme has argued that generative AI can tackle both structure prediction and sequence design in tandem, opening the door to the creation of new proteins with tailored function [46]. However, for protein systems with non-canonical glycosylation modifications or unusual glycoforms, whether these models can generalize to modified residues, glycan contexts, and non-standard structural features remains a key question that has yet to be resolved.

#### 4.3.2. AI-Assisted Cryo-EM Structure Determination

Cryo-electron microscopy (cryo-EM) has become an indispensable technique for resolving the structures of large glycoprotein complexes, yet the inherent flexibility of glycan chains and the resulting conformational heterogeneity still constrain the resolution that can be achieved. In recent years, a series of AI methods, exemplified by ModelAngelo, have begun to integrate cryo-EM density maps with protein sequence and structural information, enabling the automated building of atomic models directly into the density. For glycoproteins, AI-assisted cryo-EM is of particular value. Because glycan chains are highly dynamic, their density signals are often weak, making it difficult for traditional approaches to accurately assign their spatial positions. Protein backbones and conformational states from AI can act as priors, thereby improving particle sorting, structural refinement, and the interpretation of glycan–protein interfaces.

In the HKU1 complex, the bound sialoglycan is well resolved and its 9-O-acetyl-dependent contacts can be read directly from the map; in the OC43-C2 complex at 1.89 Å, the locally refined D1 domain similarly resolves the carbohydrate-binding site; in the Env–Fab complex at 3.7 Å, by contrast, only the core GlcNAc residues of most N-glycans are visible. This contrast defines both the reach and the current limits of cryo-EM for glycan visualization—and hence precisely where AI-based model building is most needed.

Moving forward, the combination of AI and cryo-EM is expected to achieve automated structure determination of entire glycoprotein complexes directly from experimental density data, providing a higher-resolution structural foundation for investigating the regulatory mechanisms of glycosylation.

#### 4.3.3. HDX-MS and Multiscale Experimental Validation

Although AI models can deliver high-resolution structural predictions, their outputs still demand experimental validation, particularly when it comes to dynamic conformations and flexible regions. López-Sagaseta and Urdiciain have shown that AI-predicted structures may exhibit spatial arrangement biases in complex multi-domain systems, emphasizing the need to evaluate such predictions against experimental data [47].

Hydrogen–deuterium exchange mass spectrometry (HDX-MS) provides residue-level information on local flexibility and solvent accessibility in solution. When paired with AI-predicted structures, it can be used to check whether the predicted conformations accurately reflect dynamic solution states and to uncover model limitations in flexible regions and at glycosylation-related sites.

Future glycoprotein structural studies thus demand a closed-loop workflow—spanning AI prediction through experimental validation and culminating in model refinement. Integrating experimental data from HDX-MS, cryo-EM, NMR, and other techniques allows AI predictions to be progressively refined under experimental constraints. Published examples in which AI prediction, HDX-MS, cryo-EM, and NMR are integrated for a single glycosylated system remain rare; most current workflows combine two or three of these methods rather than all four.

### 4.4. AlphaFold3 and the Structural Basis of Glycosylation Enzymes

**Figure 4:**
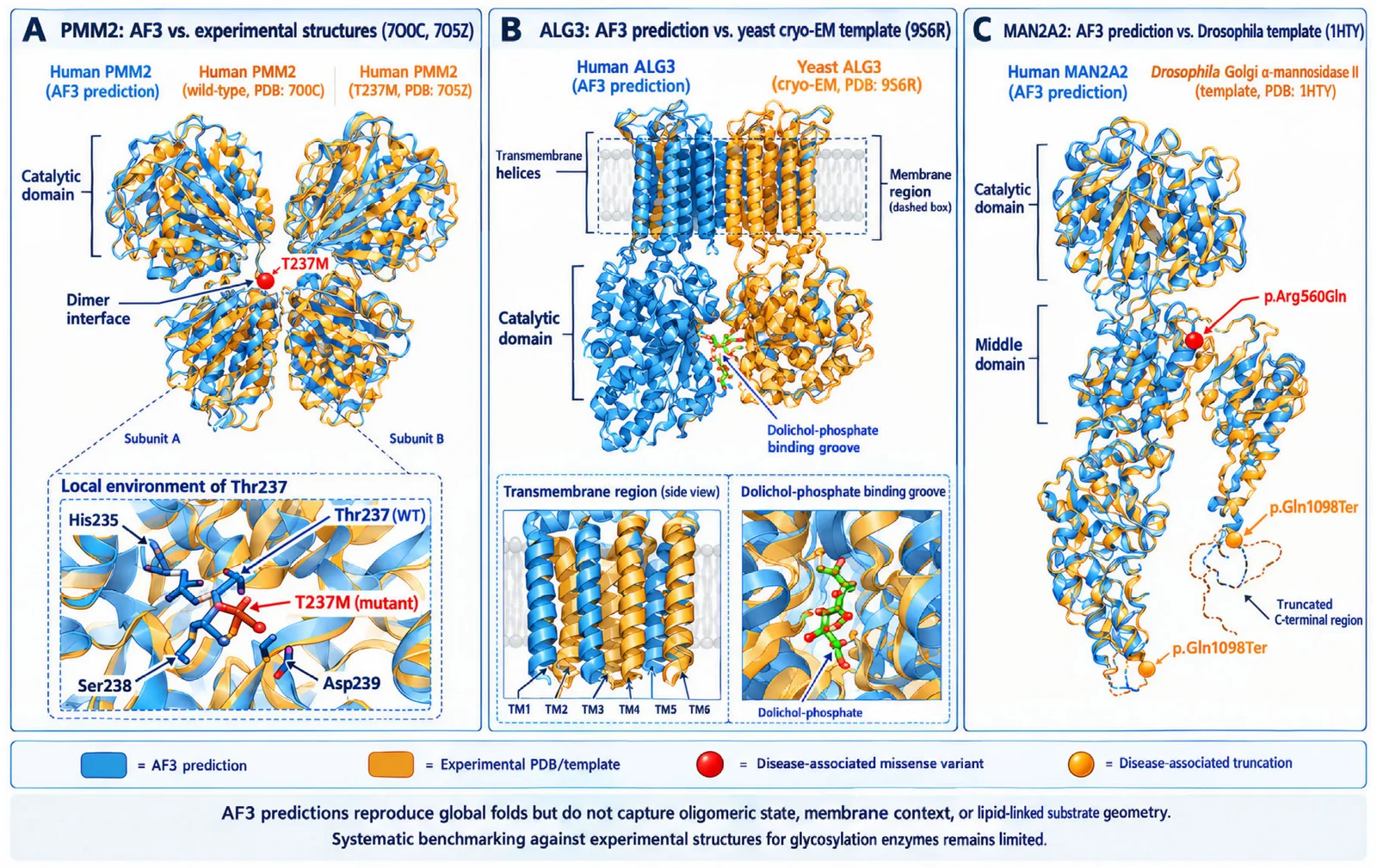
AlphaFold3 predictions compared with experimental or template structures for three glycosylation enzymes. Predicted models are shown in blue (panel B colored by pLDDT); reference structures are shown in orange. In all panels, glycan input was specified using Chemical Component Dictionary building blocks linked by bondedAtomPairs. (**A**) PMM2: AlphaFold3 reproduces the overall fold and places Thr237 at the position observed in the wild-type crystal structure (PDB 7O0C; T237M variant, PDB 7O5Z). Catalytic and cap domains, the dimer interface, and the local environment of Thr237 are indicated. (**B**) ALG3: the AlphaFold3 model of human ALG3 is compared with the yeast homolog in complex with dolichol-linked donor and acceptor (PDB 9S6R); no human structure is available, and the transmembrane helices and dolichol-binding groove are less well defined. (**C**) MAN2A2: the AlphaFold3 model is shown against Drosophila Golgi α-mannosidase II (PDB 1HTY), the template used to map p.Arg560Gln and the p.Gln1098Ter truncation.

To assess how AI-based structural prediction can inform glycosylation mechanism and disease variant interpretation, we compared AlphaFold3 (AF3) predictions with experimentally determined structures for representative enzymes acting at distinct steps and compartments of the N-glycosylation pathway (Figure 4).

PMM2 acts in the cytosol, catalyzing the interconversion of mannose-6-phosphate and mannose-1-phosphate. Crystal structures of wild-type human PMM2 (PDB: 7O0C, 2.8 Å) and the recurrent T237M variant (PDB: 7O5Z, 2.07 Å) are available [48]. AF3 reproduces the overall PMM2 fold and places Thr237 at the position seen in the wild-type crystal structure (PDB: 7O0C), correctly identifying it as a residue whose substitution is structurally consequential. The loss-of-function mechanism, however, comes from the experimental variant structure (PDB: 7O5Z): such models do not capture the dimerization equilibrium or the conformational consequences of interfacial mutation, so static predictions can guide variant interpretation but cannot replace experimental assessment of stability effects. ALG3 is an ER membrane-embedded dolichol-linked mannosyltransferase. The yeast ALG3 homolog has been resolved as a ternary cryo-EM complex with Dol25-PP-GlcNAc_2_Man_5_, Dol25-P-Man, and a Fab chaperone (PDB: 9S6R, 3.08 Å) [49]; no human ALG3 structure is yet available, so patient variants must be mapped onto the yeast template through homology—precisely the kind of task for which AI-based modeling is best suited. AF3 modeling of ALG3 without the Fab yields a catalytic domain with plausible topology, but the transmembrane helices and the dolichol-phosphate binding groove are less well defined, consistent with the general difficulty of modeling membrane-embedded glycosyltransferases and lipid-linked substrates. MAN2A2 acts in the Golgi and has no experimental structure. AF3 predictions of the wild-type enzyme align with the crystal structure of the closely related Golgi α-mannosidase II from *Drosophila* (PDB: 1HTY) [50], providing a template for mapping disease-associated variants onto the catalytic and middle domains. Here AF3 fills a genuine gap: no experimental structure exists, yet the model supports variant interpretation. *p.Arg560Gln* localizes to the α-mannosidase middle domain essential for catalytic activity, whereas *p.Gln1098Ter* truncates the C-terminal region; together, these observations are consistent with the experimentally observed accumulation of immature and hybrid-type N-glycans in serum [51]. This is precisely the scenario in which AI-based structure prediction has its greatest practical value for CDG—prioritizing variants in genes that lack experimental structures. These three cases illustrate both the promise and the limits of AF3 for glycosylation enzyme analysis. For PMM2 and ALG3, experimental structures already exist and serve as benchmarks; AF3 agrees on global fold but does not capture oligomeric state, membrane context, or lipid-linked substrate geometry. For MAN2A2, where no experimental structure is available, AF3 provides a first structural hypothesis that can be tested by biochemical and glycomic assays. The appropriate use of AF3 in CDG research is therefore not as a replacement for experimental structure determination but as a variant-prioritization and hypothesis-generation tool, particularly for the many glycosylation genes that remain structurally uncharacterized. Systematic benchmarking of AF3 against experimental structures for glycosylation enzymes is still lacking; the three cases above are illustrative rather than a controlled comparison. The clinical context for these enzymes is given in Section 5.4. And Table 1 summarizes the main structure-prediction and structure-modeling approaches discussed in Section 4.1, Section 4.2, Section 4.3 and Section 4.4 (Table 1).

### 4.5. Challenges and Future Directions

Despite the substantial progress driven by AI-based structure prediction, glycoprotein research in complex systems still faces three major challenges related to data availability, conformational dynamics, and the transition from prediction to functional design.

First, insufficient training data and restricted model generalizability still represent the main bottleneck. Existing structure databases are markedly biased in terms of species coverage, conformational states, and types of post-translational modifications. Glycosylated regions, which frequently show high evolutionary diversity and insufficient homologous sequence information, pose additional difficulties for approaches depending on multiple sequence alignments. Varadi et al. have pointed out that the resource distribution within the AlphaFold database is uneven, a bias that may compromise performance on specialized protein systems [52]. High-quality glycoprotein structural databases—annotated with glycan composition, linkage details, spatial conformations, and experimental data—thus play a critical role in improving model reliability.

Second, modeling dynamic conformations and flexible regions remains a core difficulty. Most structures in the AlphaFold database represent single dominant conformations, yet proteins in the physiological environment function as conformational ensembles [35], and the high flexibility of glycans in glycoproteins accentuates this gap. Agarwal and McShan have shown that while low-pLDDT regions can indicate flexibility, they cannot reliably distinguish functional dynamics from genuine disorder, and they emphasize that predicted models should be integrated with experimental data from small-angle X-ray scattering (SAXS), solution NMR, cryo-EM, and X-ray crystallography rather than treated as ground truth [53]. Because pLDDT and PAE are confidence rather than accuracy measures, agreement with experimental or simulated ensembles must additionally be assessed using RMSD or related comparison metrics.

Third, the shift from structure prediction to functional design is still an open frontier. Generative AI and protein language models are driving protein engineering from structural interpretation toward rational design. Standardized benchmarks for mutation-effect prediction exist [45], but their coverage of glycosylated proteins remains limited. Thus, for glycoproteins, computational tools can forecast how modifications at glycosylation sites influence local conformation and glycan accessibility, thereby supporting the rational design of therapeutic proteins and industrial enzymes.

Progress will depend on integrating multimodal AI, generative models, and experiments. Evolutionary priors come from protein language models, spatial constraints from structure predictors, and dynamic and physical feedback from MD, cryo-EM, NMR, and HDX-MS. Closed-loop cycles of prediction, validation, and refinement are the most plausible route toward functional glycoprotein design, but none of the components is yet sufficient on its own. AI’s capacity to explore uncharted structural space also offers opportunities to probe the “structural dark matter” inaccessible to conventional approaches.

## 5. AI-Driven Big Data Mining in Glycomics

The systematic interpretation of glycomics data is fundamental to understanding the regulatory mechanisms of glycosylation and its links to disease. Glycomics data, however, show substantially greater structural complexity and biological heterogeneity than genomics and proteomics data. Glycans do not possess the linear template found in nucleic acids and proteins; instead, their synthesis depends on multiple glycosyltransferases, glycosidases, and cellular factors working in concert. The branching structures, linkage isomers, and dynamic conformations of glycans generate a structural space that conventional linear biopolymers cannot easily accommodate. Extracting reliable structural information from large-scale glycomics data and then deciphering its functional significance has therefore been a long-standing core challenge in glycobiology.

In recent years, advances in artificial intelligence have provided a new computational framework for glycomics data analysis. Through deep learning, graph neural networks, and multimodal data fusion, AI can capture latent patterns in complex, high-dimensional, and nonlinear glycomics data, gradually shifting the field away from traditional database matching and manual interpretation toward data-driven structure prediction and mechanism discovery.

### 5.1. Characteristics of Glycomics Data and Challenges for AI Applications

The complexity and heterogeneity of glycomics data pose a specific set of challenges for AI.

Glycan structural complexity poses the main limitation. Nucleic acids and proteins follow linear sequences, whereas glycans are built from monosaccharide units linked via various linkages and branching patterns. Their information content is thus encoded not only in compositional differences but also in linkage positions, stereochemistry, and three-dimensional conformation. Djalali et al. have pointed out that glycan function is intimately tied to complex spatial conformations, making the information dimensionality far higher than that of linear biopolymers [54]. Ben Faleh has emphasized that the immense diversity of glycan isomers is the primary technical bottleneck for accurate structural elucidation, as the fragmentation information from conventional tandem mass spectrometry is often insufficient to uniquely assign a structure [55]. An additional challenge is that glycan biosynthesis, being template-free, relies on the coordinated activities of glycosyltransferases and glycosidases, which imposes stringent demands on chemical selectivity in both synthesis and identification. Akune-Taylor et al. further noted that glycosylation is influenced by enzyme expression, subcellular localization, substrate competition, and the cellular environment, such that the same protein can display distinct glycoforms under different conditions [56]. This phenomenon, commonly termed glycosylation microheterogeneity, adds to analytical complexity and calls for high specificity in biomarker studies.

Data non-standardization poses another major barrier. The absence of uniform representation across experimental platforms, analytical pipelines, and database systems impedes large-scale data integration and model training. Bojar and Lisacek have observed that glycoinformatics has long suffered from insufficient database scale and inconsistent representations [57] (Table 2), and the size and standardization of glycomics resources notably lag behind those of genomics and proteomics. Furthermore, glycomics data are compositional by nature. Bennett pointed out that the relative abundances of glycoforms sum to a constant, violating the independence assumptions of conventional statistical methods; log-ratio transformations are therefore required to provide a sound data foundation for machine learning [58]. Table 2 summarizes representative glycomics data resources and their limitations.

In summary, the core challenge in glycomics lies in extracting biological meaning from high-dimensional, heterogeneous data. The strength of AI resides in its capacity to learn patterns that are difficult to capture with traditional approaches, yet its effective application hinges critically on data quality, annotation consistency, and the incorporation of glycan-specific chemical priors.

### 5.2. AI-Driven Glycan Structure Elucidation and Mass Spectrometry Data Mining

Mass spectrometry serves as the core data-generation tool in glycomics, but traditional spectral interpretation relies heavily on database matching and expert curation, leaving it ill-equipped to handle isomeric mixtures and unknown structures. Deep learning is now reshaping this paradigm by substantially reducing dependence on predefined candidate libraries.

CandyCrunch, developed by Urban et al., employs a dilated residual neural network trained on over 500,000 annotated mass spectra and predicts glycan structures directly from raw spectra without requiring a candidate list, automatically learning fragmentation rules and structure–spectrum relationships [25]. Even with low signal-to-noise data, the model works well, yet its accuracy falls considerably for rare glycoforms, non-canonical modifications, and highly branched glycans—a shortcoming that points to limited training-data coverage and its consequences for generalizability. In a complementary approach, Yang Yi and Fang Qun proposed DeepGlyco, which combines Tree-LSTM and graph neural networks to build a forward prediction framework that simulates the fragmentation patterns of different glycopeptides, thereby providing theoretical reference spectra for experimental data [59]. CandyCrunch and DeepGlyco represent reverse and forward prediction strategies, respectively; integrating the two could create a closed computational simulation loop that effectively reduces false positives.

A concrete test case is provided by immunoglobulin G (IgG). Core fucosylation, galactosylation, and sialylation of the conserved Fc N-glycan each shift effector function, and deep learning applied to released-glycan and glycopeptide profiles has been used to resolve Fc glycoform distributions and to associate them with autoimmune and malignant phenotypes [60].

A complementary computational framework, GlyCompute, addresses a different bottleneck: the kinetic modeling of N-glycosylation. GlyCompute automates the assembly, simulation, and parameterization of kinetic models of protein N-linked glycosylation from experimentally observed glycan structures and their relative abundances. Reaction networks are generated as directed acyclic graphs and used to assemble the material balances and kinetic mechanisms automatically, and parameters are estimated by a sequential Bayesian strategy whose stages are determined from network topology. Tested on recombinant IgG and host-cell proteins from the same CHO culture, the framework reconciled both glycoprofiles with one parameter set [61]. The current version does not address glycan structural ambiguities such as linkage uncertainty, and the approach has been demonstrated on a single case study.

The representation learning of glycans is equally critical. Joeres, Bojar, and colleagues employed graph neural networks, treating monosaccharides as nodes and glycosidic bonds as edges, and used message passing to capture topological features, breaking through the constraints of linear representations [20]. Kitani et al. later introduced GlycanGT, which brings a Graph Transformer architecture to glycan foundation models. Through large-scale self-supervised pretraining, GlycanGT learns long-range dependencies and has demonstrated capabilities in unknown structure completion and functional prediction, suggesting that the modeling paradigm of protein language models can be transferred to glycans [62]. That said, the scale of pretraining data remains far smaller than that in the protein domain, three-dimensional conformational information has yet to be incorporated, and the gains for downstream tasks still require further validation.

Although AI has markedly strengthened structural elucidation, its performance continues to be constrained by the scope of the training data. Future efforts should aim at expanding high-quality annotated datasets and integrating ion mobility mass spectrometry, molecular dynamics simulations, and multimodal fusion to improve the capacity to characterize unknown structures and dynamic behavior.

### 5.3. Multimodal Data Fusion Advances Functional Interpretation of Glycosylation

While glycomics catalogs the structures and abundances of glycans, glycan function relies on intricate links with protein expression, the cellular milieu, and disease—links that a single data type is insufficient to fully unravel. Multimodal AI offers a framework for integrating glycomics, glycoproteomics, and other omics layers, shifting research from structural identification toward functional elucidation.

The central challenge in multi-omics integration is data heterogeneity. Conventional integration methods struggle to cope with high-dimensional sparsity and nonlinear relationships. Baião et al. noted that deep generative models, by jointly embedding multiple data types in a shared latent space, can effectively manage batch effects and missing values, laying a foundation for cross-omics association [63]. However, such models remain limited in interpretability. Latent-space dimensionality and prior selection can both strongly affect results, which necessitates biological priors serving as constraints. Yazdani and coworkers proposed a network-based AI integration approach that builds association networks across omic strata and employs causal inference to pinpoint key regulatory modules, thus uncovering nonlinear connections among glycosyltransferase expression, metabolic reprogramming, and glycoform changes [64].

In disease contexts, machine learning approaches can already identify glycoform signatures in serum glycan profiles that correlate with immune disorders and tumors, which aids molecular subtyping and biomarker discovery. Nevertheless, current classifiers offer little mechanistic understanding, and their reproducibility in independent cohorts remains to be established.

Congenital disorders of glycosylation (CDG) represent the complementary case in which the causal lesion lies in the glycosylation machinery itself: pathogenic variants in glycosyltransferase, glycosidase, and sugar-nucleotide transporter genes produce predictable defects in N- and O-glycan processing. Here AI contributes at two levels—structure-aware models help prioritize variants of uncertain significance by locating them within catalytic or substrate-binding regions, while classifiers trained on serum transferrin or total released glycans support screening and subtyping [65].

Multimodal AI is shifting glycomics from merely “describing changes” toward “deciphering regulatory networks”. Yet most studies are still confined to correlative observations; whether glycosylation acts as a downstream consequence or an upstream driver remains to be disentangled through causal inference and experimental validation.

### 5.4. Congenital Disorders of Glycosylation as a Translational Test Case

Congenital disorders of glycosylation (CDG) are a group of rare, multisystemic genetic diseases caused by defects in glycan biosynthesis and protein glycosylation. They provide the clearest example of how a defect in a ubiquitous cellular pathway translates into human disease. Clinically, CDG typically presents with neurological involvement, hepatic dysfunction, failure to thrive, and variable immune and endocrine abnormalities. The molecular lesions span the entire glycosylation pathway: *PMM2* encodes phosphomannomutase 2, which converts mannose-6-phosphate to mannose-1-phosphate, a precursor for GDP-mannose and dolichol-linked oligosaccharide synthesis; *PMM2-CDG* is the most common single genotype, accounting for the majority of diagnosed cases [66]. *ALG3* encodes a dolichol-linked mannosyltransferase that acts early in the ER lumen during lipid-linked oligosaccharide assembly. More recently, *MAN2A2*, which encodes a Golgi α-mannosidase required for N-glycan trimming and maturation, has been identified as a candidate gene in autism spectrum disorder and cognitive delay: biallelic variants (p.Arg560Gln and p.Gln1098Ter) produce accumulation of immature and hybrid-type N-glycans in serum [51].

AI-driven approaches are beginning to contribute to CDG research at several levels. For variant interpretation, structure-aware models help prioritize variants of uncertain significance in glycosylation genes by integrating structural context with molecular dynamics simulations. For biomarker discovery, machine-learning models applied to circulating miRNA profiles have shown promise in PMM2-CDG: an XGBoost classifier achieved an AUC of 0.917, and miR-122-5p emerged as the most highly weighted marker, indicating liver and neurological involvement [67]. For glycomic classification, machine-learning classifiers trained on glycan profiles have been applied to distinguish CDG subtypes and group patients by glycan or proteomic similarity, complementing classical transferrin isoelectric focusing and aiding the refinement of ambiguous cases [65]. Structural models for these enzymes, and their agreement or disagreement with available experimental structures, are discussed in Section 4.4.

### 5.5. Explainable AI Facilitates the Discovery of Glycobiological Mechanisms

Mere predictive accuracy is insufficient for the demands of biology. For AI to transition from a computational tool to a genuine mechanism-discovery platform, we must grasp the molecular reasoning behind model predictions and derive testable rules from data. Explainable AI (XAI) fulfills exactly this role: via feature attribution, concept extraction, and counterfactual reasoning, it turns black-box outputs into testable biological hypotheses.

In glycobiology, XAI can be applied at two levels: identifying novel regulatory factors from omics data and inferring causal relationships between glycosylation modifications and phenotype.

At the level of regulatory factor discovery, Qin Rui et al. incorporated SHapley Additive exPlanations (SHAP) into single-cell glycosylation prediction to pinpoint key genes that shape glycan branching. These included not only established regulators but also low-abundance factors that tend to be overlooked by conventional differential analysis because they are eliminated by stringent multiple hypothesis correction; AI captured them through joint interaction effects [68]. The utility of XAI in helping researchers formulate hypotheses and direct subsequent experiments is illustrated here.

A recent example extends explainable AI to high-throughput glycan profiling. LeGenD combines lectin-binding data with explainable AI to predict dominant N-glycan structures and their relative abundances on purified proteins [69]. The model was trained on 309 glycoprofiles from 10 recombinant proteins produced in 30 glycoengineered CHO cell lines, and SHAP analysis identified the lectins that most strongly influence prediction outcomes, providing interpretable links between accessible lectin-binding measurements and underlying glycan structures [69]. This work illustrates how XAI can bridge the gap between routine experimental readouts and the detailed structural information required for bioprocess monitoring and clinical diagnostics.

At the heart of causal inference lies the enzyme–substrate specificity that underpins glycosylation control. Terminal glycan structures, linkage types, and protein environments are preferred differently by various enzymes—a fact that makes it hard to resolve multi-enzyme competition with classical experiments. From interaction data, AI can derive the sequence and structural cues that determine selectivity and can predict new reactions. Yet labeled datasets remain sparse and skewed toward common model organisms, constraining both reliability and generalizability. Moving from correlation to causation is a key objective of biomedical AI. When a model uncovers a strong association between a glycoform and a disease, causal methods can further determine whether the glycoform change is a trigger or an accompanying phenomenon—a distinction that is crucial for prioritizing therapeutic targets. Causal inference in glycomics is still at an early stage; the main barrier is the lack of time-series and exogenous intervention data. Future efforts will need to draw on longitudinal cohorts and CRISPR screens to supply the identifying conditions that causal models require.

Glycomics AI should not be confined to boosting predictive performance; rather, it should combine explainable models, experimental validation, and causal analysis to become a genuine mechanism discovery tool. It is important to note that the choice of explanation method should be guided by the model architecture and the specific biological question. Different attribution algorithms can produce varying outcomes, and their results require careful interpretation and cross-validation.

### 5.6. Current Challenges and Future Directions

AI has produced notable progress, yet glycomics still encounters numerous hurdles with data, modeling, and biological interpretation.

High-quality training data remain scarce. The pool of glycan structures confirmed by rigorous validation is small, and data on rare glycoforms, complex branching patterns, and unusual modifications are even more limited. The identification of many structures rests solely on mass spectral matching, without confirmation from NMR or chemical synthesis—a practice that carries the risk of false positives. Although pretraining and transfer learning can partially mitigate data scarcity, generalization to low-abundance and novel structures is still restricted. Future work should prioritize developing systematic, standardized databases, leveraging self-supervised and few-shot learning, and promoting cross-laboratory data sharing with standardized workflows.

Three-dimensional dynamic conformations are largely absent from current models. Existing approaches focus on two-dimensional topology, yet glycan function depends on dynamic three-dimensional conformations. Their interactions with proteins, antibodies, and lectins are governed by glycans’ flexible motions in solution. Most models have limited capacity for conformational sampling, and large-scale 3D conformational databases and encoding schemes are lacking. AI, combined with MD simulations, glycan-specific structure predictors, and experimental data, will push the field beyond static analysis toward dynamic functional prediction, while also relating conformational changes to biological activity in quantitative terms.

Insufficient data standardization and reproducibility hamper progress. Variations in experimental platforms, mass spectrometry parameters, and data representation schemes restrict both model training and cross-study comparisons. The MIRAGE guidelines proposed by Lageveen-Kammeijer et al. have laid a foundation for improving data quality, yet their adoption remains limited. Future work should build on standardized and open data frameworks, encouraging the release of raw data, acquisition parameters, and analysis code to enhance transparency and reproducibility [70].

The spatiotemporal dynamics of glycosylation often receive insufficient attention. Glycosylation patterns are inherently dynamic, shifting with cell state, environmental cues, and disease progression, yet most models still view them as static snapshots. Future work should incorporate time-series data and dynamic modeling to clarify how regulatory networks evolve with physiological state and to pinpoint critical nodes and early transition signals.

**Figure 5:**
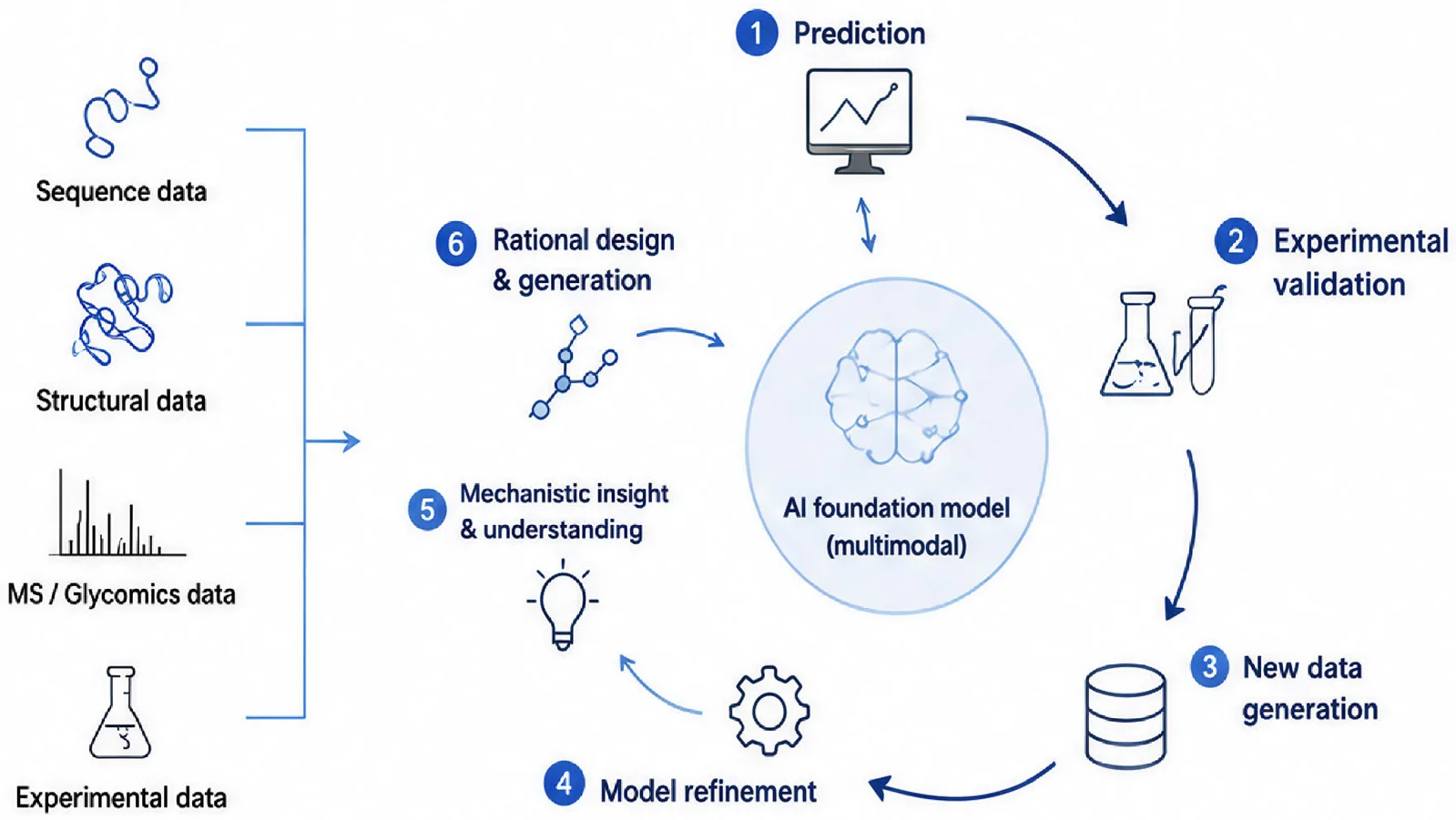
Toward generation: a closed-loop Al framework. This schematic illustrates the iterative cycle that integrates multiple data streams—protein sequences, structural information, mass spectrometry/glycomics profiles, and experimental measurements—into a multimodal AI foundation model for prediction. The model undergoes continuous refinement, generating mechanistic insights that inform rational design and glycoengineering hypotheses. These predictions are then tested through experimental validation, producing new data that are fed back into the model to further improve its performance. This closed-loop architecture embodies the shift from static prediction toward active generation, enabling data-driven discovery and the intelligent design of glycosylated molecules.

Overall, AI is moving glycomics from structural elucidation toward functional prediction and mechanistic discovery (Table 3, Figure 5). Through the integration of foundation models, multimodal learning, dynamic structural modeling, and closed-loop experimental–computational validation, glycomics AI has the capacity to surpass current barriers—advancing from structural identification to systems-level understanding of regulatory networks and ultimately offering new avenues for disease diagnosis, prognosis, and precision therapy.

## 6. Conclusions

This review has systematically surveyed recent advances in applying artificial intelligence to protein glycosylation research. Organized around four major themes—prediction of glycan synthesis, identification of glycosylation sites, structural modeling of glycoproteins, and glycomics data mining—it outlines the broader landscape of an AI-driven transformation in glycoscience. The information flow of glycosylation, spanning glycosidic bond formation, modification site selection, three-dimensional conformation, and omics-scale profiling, forms a continuous chain from chemical bonds to biological function, and AI is now laying down new methodological foundations at every link along that chain.

In glycochemistry, machine learning models have achieved quantitative prediction of glycosidic bond stereoselectivity and active optimization of reaction conditions, with GlycoPredictor and Bayesian optimization representing the two complementary paradigms of rule mining and autonomous discovery. In site identification, protein language models and graph neural networks have endowed predictors with structure-aware capabilities that reach beyond sequence motifs, markedly improving recognition of non-canonical and low-abundance modification sites. In structural biology, static snapshots of glycoprotein structures are giving way to dynamic conformational ensembles through the AlphaFold series and multimodal learning. In glycomics, end-to-end deep learning combined with explainable AI is enabling high-throughput glycan structure elucidation while probing the regulatory logic that underlies it.

A unifying thread across these developments is the ability of AI to extract distribution patterns and structural constraints from high-dimensional, heterogeneous glycan data—patterns that have largely eluded traditional methods. High-quality annotated data are scarce, dynamic conformational encoding is missing, and physical consistency conflicts with interpretability—these three issues form the current bottlenecks. The way ahead calls for developing multimodal foundation models designed specifically for glycoscience, creating standardized data repositories with closed-loop experimental feedback, and shifting decisively from predictive tools toward true functional design capacity.

## Figures and Tables

**Table 1:** Comparison of representative structure-prediction and structure-modeling approaches.

Method	Biological System	Glycan Modeling	Conformational Dynamics	Input/Validation	Advantages/Limitations
AlphaFold2	Monomeric proteins	No	No	Sequence, MSA/experimental structures	High accuracy for folded domains; no glycan or dynamics
AlphaFold-Multimer	Protein complexes	No	No	Sequence, MSA/complex structures	Interface prediction; no glycan, limited flexibility
AlphaFold3	Proteins, nucleic acids, ligands, glycans	Partial	No	Sequence, CCD/BAP/experimental structures	Unified framework with glycan input; static, confidence metrics fail for glycans
RoseTTAFold/RFAA	Biomolecular assemblies	Partial (modified residues)	No	Sequence, atomistic context/experimental structures	All-atom coverage; glycan parameterization limited
DeepGlycanSite	Carbohydrate-binding proteins	Yes (binding site)	No	Structure, evolution/benchmark datasets	High binding-site accuracy (MCC 0.625); not a full glycoprotein model
AlphaFLOW	Proteins (monomers and complexes)	No	Yes	AlphaFold structures/MD, reference ensembles	Generative sampling; not glycan-specific
AI-assisted cryo-EM	Glycoprotein complexes	Yes (density)	Partial	Density maps, sequence/PDB validation	Resolves ordered glycan density; limited at low resolution

“Glycan modeling” indicates whether glycan chains are represented in the model input or output. “Conformational dynamics” indicates whether the method samples multiple conformations rather than producing a single static structure. “Partial” indicates that glycans or modified residues are handled only to a limited extent. Quantitative glycoprotein-specific benchmarks are not yet available for most methods; where performance on glycoproteins has not been reported, this is indicated in the table. The absence of a shared glycoprotein benchmark is itself a limitation of the current literature.

**Table 2:** Representative glycomics data resources and their limitations.

Resource	Content and Data Type	Scale	Standardization/QC	Key Limitation
GlyTouCan	International glycan structure repository providing unique accession numbers and standardized structural encoding	~250,000 registered glycan structures	Community-driven curation; standardized structural encoding	Structure-linked annotations (linkage, stereochemistry) often incomplete; coverage uneven across glycan classes
GlyConnect	Curated glycan–protein associations and glycosite information with species, tissue, and disease context	~22,600 glycosylation sites on ~2200 glycoproteins; ~5600 glycan structures	Expert curation from the literature; preserves biological context	Uneven coverage across glycosylation types; cross-resource identifier mapping inconsistent
UniCarbKB	Glycan structures with associated mass spectra and glycoprotein information	~1530 glycoprotein entries; >4000 annotated glycosylation sites	Curated structural and experimental data; integrates HPLC, UPLC, exoglycosidase sequencing, and MS	Database scale and standardization lag behind genomics/proteomics; updates have been limited
GlycoPOST	Raw and processed mass spectrometry data repository for glycomics	73 datasets (~2000 files, ~700 GB) as of 2023	Metadata follows MIRAGE guidelines; FAIR-compliant	Raw-data coverage still small relative to proteomics repositories; completeness depends on submitter compliance
GlyGen	Multi-omics knowledgebase linking glycan, protein, gene, and disease data	58,841 glycans; 243,702 proteins; 183,573 glycosylation sites across 16 organisms	Harmonized data model integrating multiple ontologies; extensive cross-references	Coverage of glycosylation types and species remains incomplete; 3D conformational data largely absent
MIRAGE	Reporting guidelines for glycomics experiments (sample preparation, MS, LC, glycan/lectin microarray, CE, and NMR)	Multiple guideline documents	Community-developed minimum information standards; hosted by Beilstein-Institut	Adoption is uneven across laboratories and journals; compliance is voluntary

This table summarizes representative glycomics data resources and their limitations. Resources were selected for their widespread use and coverage of glycan structures, glycoproteins, glycosites, and related metadata; scale values are approximate, compiled from official statistics or primary publications. MIRAGE is a reporting guideline rather than a database. The limitations reflect gaps that currently constrain large-scale AI training and benchmarking. Abbreviations used in this table are defined as follows: HPLC, high-performance liquid chromatography; UPLC, ultra-performance liquid chromatography; MS, mass spectrometry; LC, liquid chromatography; CE, capillary electrophoresis; NMR, nuclear magnetic resonance; MIRAGE, Minimum Information Required for a Glycomics Experiment.

**Table 3:** Representative AI methods, algorithms, and benchmarks for protein glycosylation research.

Method/Algorithm	Input	Output/Benchmark	Key Limitation
GlycoPredictor (decision tree + random forest)	Donor, acceptor, protecting groups, solvent, temperature	Anomeric selectivity (α/β ratio); AUC 0.89–0.97	Evaluated on ~10,434 reactions (CAS, 2010–2015); prospective validation uncommon; cross-publication R^2^ drops to 0.11
Bayesian optimization (Faurschou & Pedersen)	Reaction conditions (no prior data)	Optimal conditions; lithium-salt-directed glycosylation	Demonstrated on single reaction type; generality across substrate classes not established
N-GlycoPred (CNN + BiLSTM)	Protein sequence	N-glycosylation sites; reported sensitivity 95.56%, specificity 96.56%, accuracy 96.30%, MCC 0.9066; average AUC 0.9553 across six independent test datasets	Performance not directly comparable across studies due to different negative sets; independent external validation uncommon
HOTGpred (PLM features + XGBoost)	Protein sequence + pretrained PLM embeddings	O-linked threonine glycosylation sites; improved accuracy relative to earlier sequence-based predictors	O-glycosite prediction lacks universal consensus motif; cross-study comparison limited
PTG-PLM	Protein sequence (PLM embeddings)	Post-translational glycosylation and glycation sites	First PLM application to glycosylation; evaluation on author-assembled datasets
EMNGly	Protein sequence (PLM features)	N-glycosylation sites	Limited cross-study comparison due to different negative-set construction
CoNGlyPred (ESM-2 + GNN + co-attention)	Sequence + AlphaFold2-predicted structural features	N-glycosylation sites	Structural features derived from predicted (not experimental) structures; error propagation not systematically assessed
AlphaFold2, AlphaFold3, RoseTTAFold/RFAA, DeepGlycanSite, AlphaFLOW	Sequence, MSA, CCD/BAP, structural and evolutionary features	See Table 1 (Section 4.4) for systematic comparison	—
CandyCrunch (dilated residual CNN)	Raw LC-MS/MS spectra	Glycan structures; top-1 accuracy 90.3%	Multiclass classifier limited to training-set structures; accuracy drops for rare glycoforms
DeepGlyco (Tree-LSTM + GNN)	Glycopeptide composition	Theoretical reference spectra; forward prediction	Complementary to CandyCrunch; integration not yet demonstrated at scale
GlycanGT (Graph Transformer)	Glycan graph	Structure completion; functional prediction	Pretraining data far smaller than protein domain; three-dimensional conformational information has yet to be incorporated
GlyCompute (Bayesian parameter estimation, ABC-SMC)	Observed glycan structures + abundances	Kinetic model parameters; glycoprofile prediction	Does not address linkage uncertainty; demonstrated on single case study
LeGenD (SHAP + lectin profiling)	Lectin-binding data	N-glycan structures + relative abundances	Trained on 30 glycoengineered CHO cell lines; generalizability to other systems not established

This table summarizes representative AI methods, algorithms, and benchmarks across the four application areas covered in this review. It is not exhaustive; methods were selected for their prominence and relevance to glycosylation research. Performance metrics are as reported in the original studies. Structure-modeling approaches are compared in detail in Table 1.

## Data Availability

No new data were created or analyzed in this study. Data sharing is not applicable to this article.
